# Retraction: Mathematical Modelling and Prediction of the Effect of Chemotherapy on Cancer Cells

**DOI:** 10.1038/srep46986

**Published:** 2018-06-01

**Authors:** Hamidreza Namazi, Vladimir V. Kulish, Albert Wong

Scientific Reports 5: Article number: 1358310.1038/srep13583; published online: 08 28 2015; updated: 06 01 2018

This Article has been retracted by *Scientific Reports* at the request of Nanyang Technological University. An investigation at Nanyang Technological University found that ethical approval for the reported experiments was not sought from their Internal Review Board.

The Authors do not agree with the Retraction.

